# Transposition Complex with Aortic Arch Obstruction: Outcomes of One-Stage Repair Over 10 Years

**DOI:** 10.1007/s00246-015-1258-6

**Published:** 2015-09-10

**Authors:** Kwang Ho Choi, Si Chan Sung, Hyungtae Kim, Hyung Doo Lee, Gil Ho Ban, Geena Kim, Hee Young Kim

**Affiliations:** Department of Thoracic and Cardiovascular Surgery, Pusan National University Yangsan Hospital, Geumo-ro 20, Beomeo-ri, Mulgeum-eup, Yangsan-si, Gyeongsangnam-do 626-770 Republic of Korea; Department of Pediatric Cardiology, Pusan National University Yangsan Hospital, Geumo-ro 20, Beomeo-ri, Mulgeum-eup, Yangsan-si, Gyeongsangnam-do 626-770 Republic of Korea; Department of Anesthesiology, Pusan National University Yangsan Hospital, Geumo-ro 20, Beomeo-ri, Mulgeum-eup, Yangsan-si, Gyeongsangnam-do 626-770 Republic of Korea

**Keywords:** Transposition of the great vessels, Double outlet right ventricle, Aortic coarctation, Interruption of aortic arch

## Abstract

The surgical management of transposition complex with aortic arch obstruction remains technically demanding due to anatomic complexity. Even in the recent surgical era, there are centers that address this anomaly with a staged strategy. This report presents our experiences with a one-stage repair of transposition complexes with aortic arch obstructions more than the last 10 years. Since 2003, 19 patients with a transposition of the great arteries (TGA, 2 patients) or a double outlet of the right ventricle (DORV, 17 patients) and aortic arch obstruction have undergone one-stage repair of their anomalies. The mean age was 6.7 ± 2.3 days, and the mean body weight was 3.4 ± 0.3 kg. The 2 patients with TGA exhibited coarctation of the aorta. The 17 patients with DORV all exhibited the Taussig–Bing type. The great artery relationships were anteroposterior in 4 patients (21.1 %). The coronary artery anatomies were usual (1LCx; 2R) in 8 patients (42.1 %). There were 2 early deaths (10.5 %). Seven patients (36.8 %) required percutaneous interventions. One patient required re-operation for pulmonary valvar stenosis and left pulmonary artery patch angioplasty. The overall survival was 84.2 %. The freedom from mortality was 83.5 % at 5 years, and the freedom from intervention was 54.4 % at 5 years. The one-stage repair of transposition complexes with aortic arch obstructions resulted in an acceptable survival rate and a relatively high incidence of postoperative catheter interventions. Postoperative catheter interventions are highly effective. Transposition complexes combined with aortic arch obstructions can be managed by one-stage repair with good early and midterm results.

## Introduction

In patients with transpositions of the great arteries (TGAs) with intact ventricular septa, comorbid aortic arch obstructions (AAOs) are rare. The natural history of this combination is very poor. In contrast, AAOs are more common in specific forms of ventriculoarterial discordance with ventricular septal defect (VSD), particularly in cases with double outlet right ventricle with subpulmonary VSD (Taussig–Bing anomaly) in which the incidence of AAO is approximately 50 % [9]. In the past, the surgical approach to this complex was a two-staged repair that included primary coarctation repair with or without pulmonary artery banding followed by an arterial switch and intracardiac repair after some months. However, this approach has resulted in high hospital mortality rates of 31–64 % [11, 19]. The main causes of this high mortality are the presence of tubular hypoplasia of aortic arch in many patients, the deleterious effects of pulmonary artery banding, and other factors. With the development of neonatal cardiac surgery, single-stage repair for these patients has been adopted as an alternative surgical strategy by experienced centers after it was first described by Pigot et al. [10]. However, the surgery can be complicated by various coronary artery anatomies, size discrepancies between the great arteries and the presence of the subaortic obstructions. Even in the recent surgical era, some surgeons are concerned about complicated procedures and future right ventricular obstructions and thus address these anomalies using a staged strategy. This report presents our experiences with one-stage repairs of transposition complexes with aortic arch obstructions over more than the last 10 years.

This study included all patients who underwent a single-stage repair of a TGA or TBA with AAO at our center between October 2003 and March 2015 and focused on mid- to long-term outcomes.

## Materials and Methods

### Patients

Between October 2003 and March 2015, 19 patients underwent arterial switch operations (ASO) with simultaneous repair of an aortic arch obstruction. There were 13 boys and 6 girls. The mean age at the time of operation was 6.7 ± 2.3 days. The mean body weight of these patients was 3.4 ± 0.3 kg.

There were 2 patients with TGA with VSD and 15 patients with TBA. Intravenous prostaglandin infusion was necessary for all patients to maintain the patency of the ductus arteriosus, and 3 patients required preoperative ventilator care. Six patients (31.6 %) had interrupted aortic arches, and the others exhibited coarctation of the aorta (CoA). The great artery relationships were anteroposterior in 4 patients (21.1 %) and side by side in 15 patients. The coronary artery anatomies were typical (1LCx; 2R) in 8 patients (42.1 %) and single sinus coronary anatomies in 5 patients. Two patients (10.5 %) had intramural coronary arteries, and another 6 patients (31.6 %) had retropulmonary coronary arteries. The precise coronary artery patterns are depicted in Table 1.

**Table 1:** Preoperative patient data

Variables	Patients number	%
Age (mean ± SD) days	6.7 ± 2.3	
Body weight (mean ± SD) kg	3.4 ± 0.3	
Gender		
Male	13	68.4
Female	6	31.6
Diagnosis		
TGA with VSD	2	10.5
Taussig–Bing	17	89.5
Arch anomaly		
Coarctation of aorta	13	68.4
Interruption	6	31.6
Type A	2	10.5
Type B	4	21.1
Coronary artery pattern		
1L,Cx; 2R	8	42.1
1L; 2Cx,R	2 (1RP)	10.5
1R; 2L,Cx	3 (2RP)	15.8
1R,L; 2Cx	1 (RP)	5.3
2L,Cx,R	1 (IM)	5.3
2L,Cx; 2R	3 (1RP, 1IM)	15.8
2R; 2L,Cx	1 (RP)	5.3
Relation of gas		
Anteroposterior	4	21.1
Side by side	15	78.9
PGE1	19	100
Mechanical ventilation	3	15.8

*TGA* transposition of great arteries, *VSD* ventricular septal defect, *LCA* left coronary artery, *L* left anterior descending coronary artery, *Cx* left circumflex coronary artery, *R* right coronary artery, *RP* retropulmonary coronary artery, *IM* intramural coronary artery, *GAs* great arteries, *PGE1* prostaglandin E1

### Surgical Technique

All operations were performed with a routine open heart surgical technique. Myocardial protection was performed via intermittent infusions of a 1:1 coldblood cardioplegic solution into the aortic root or coronary ostia every 30 min. Additional ductal cannulation for distal perfusion was performed in cases of interruptions of the aorta or severe coarctation. We closed the VSD before arch reconstruction and coronary transfer. The VSD was closed through the tricuspid valve in 17 patients (89.5 %) and through the tricuspid valve with right ventriculotomy in 2 patients. During the aortic arch reconstruction, we used regional perfusion via the innominate artery using a Gore-Tex tube graft (Gore-Tex^®^ Vascular graft; W.L. Gore & Associates, Inc. Flagstaff, AZ, USA). The ductal tissue was completely excised. The divided small distal ascending aorta and aortic arch were reconstructed with glutaraldehyde-treated autologous pericardial patches that were adjusted for the size of the large proximal main pulmonary artery. The coronary artery transfer technique utilized in our center has previously been described [2]. The coronary arteries were transferred to the neoaorta after completion of the neoaorta and aortic arch reconstructions. The defects of the neopulmonary artery were reconstructed with two generous fresh autologous pericardial patches. We utilize a very low threshold for sternal opening in the immediate postoperative period. All but one patient were transferred to the intensive care unit with an open sternum. We typically closed the sternum on the second postoperative day. A peritoneal dialysis catheter was routinely placed in the abdominal cavity. All of these operations were performed by a single surgeon in our center. The cardiopulmonary bypass time for the surgical procedure and the aortic cross-clamp times are listed in Table 2.

**Table 2:** Perioperative patient data

Variables	
CPB time (mean ± SD)	286.3 ± 37.3 m
ACC time (mean ± SD)	180.8 ± 21.8 m
Regional perfusion time (mean ± SD)	55.2 ± 4.9 m
VSD closure	
TV (n/%)	17 (89.5)
TV + RV tomy (n/%)	2 (10.5)
Open sternum (n/%)	18 (94.7)
Ventilator time (mean ± SD)	4.2 ± 1.8 d
Hospital stay (mean ± SD)	20.0 ± 9.8 d

*m* minutes, *d* days, *CPB* cardiopulmonary bypass, *ACC* aorta cross-clamping, *VSD* ventricular septal defect, *TV* tricuspid valve

### Postoperative Care

Inotropic support was routinely initiated with low-dose dopamine (5 µg/kg/min), and other inotropic agents, such as dobutamine and epinephrine, were selectively used. Nitroprusside was used to reduce the afterload on a case-by-case basis. Full sedation with mechanical ventilation was continued for 2 days to prevent pulmonary hypertensive crises, and peritoneal dialysis was used to remove water from the body when needed. The ventilation time was 4.2 ± 1.8 days, and the hospital stay was 20.0 ± 9.8 days.

### Statistical Analysis

The patients’ data are described as frequencies and means with standard deviations. The long-term survival rate and freedom from re-intervention were calculated using the Kaplan–Meier method. Statistical analyses were performed using Statistical Package for the Social Sciences (SPSS, version 19.0, Chicago, IL, USA). Statistical significance was indicated by a *p* value <0.05.

## Results

### Early Mortality and Morbidity

There were 2 early mortalities (10.5 %). The first was a 6-day-old and 3 kg baby with TBA with CoA, CATCH 22, a side-by-side relationship of the great artery, and a 1R,L; 2Cx coronary artery with high takeoff of the sinus 1 coronary artery. This patient had severe hepatomegaly preoperatively. The patient underwent an uneventful surgery but exhibited severe capillary leakage postoperatively. This patient died on postoperative day 3 after ECMO support. The second mortality was an 8-day-old, 2.9 kg baby with TGA/VSD and CoA, an anteroposterior relationship of the great artery, and the usual coronary artery pattern. This patient had a relatively small tricuspid valve, and the tricuspid valve was torn during the VSD closure. A tricuspid valve repair was performed, but significant tricuspid valve regurgitation continued and caused right heart failure and low cardiac output. He died on postoperative day 3.

There were early morbidities in 6 patients. One patient exhibited mild left ventricular dysfunction, one exhibited temporary arrhythmia, 2 exhibited superficial wound dehiscence, and 2 exhibited severe capillary leakage.

### Follow-up

There was one late mortality. A 6-day-old, 3.6 kg baby with TBA and type A IAA also had congenital lobar emphysema of right middle lobe. Her great artery relationship was side by side, and her coronary artery pattern was 2R; 2LCx with an intramural right coronary artery between the great vessels. These coronary arteries were transferred with an aortocoronary flap technique. Revision of aortocoronary flap was required due to persistent bleeding. The patient was weaned from the bypass with mild left ventricular dysfunction. After discharge, the pulmonary symptoms were aggravated due to right middle lobe pulmonary emphysema. A right middle lobe lobectomy was performed at 6 months postoperatively without any event. During the OPD follow-up, this patient had recurrent aspiration pneumonia and intermittent ventricular tachycardia. At 10 months postoperatively, he was transferred to the emergency room with death on arrival. The cause of death was strongly suspected to be arrhythmia.

The follow-up duration was 40.2 ± 36.9 months. Of the 16 survivors, 14 underwent complete echocardiography, and clinical follow-up data were obtained.

**Fig. 1:**
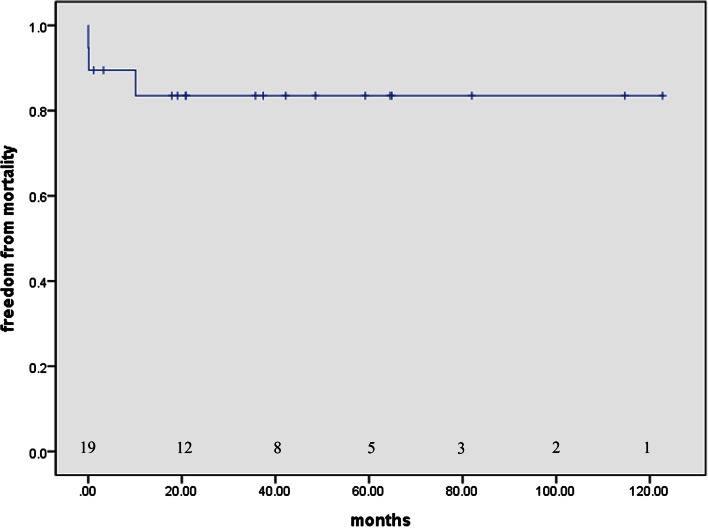
Freedom from mortality

The freedom from mortality was 83.5 % at 5 years (Fig. 1). The three deaths occurred before 2006. There have been no early or late mortalities among the 16 patients since 2006.

**Fig. 2:**
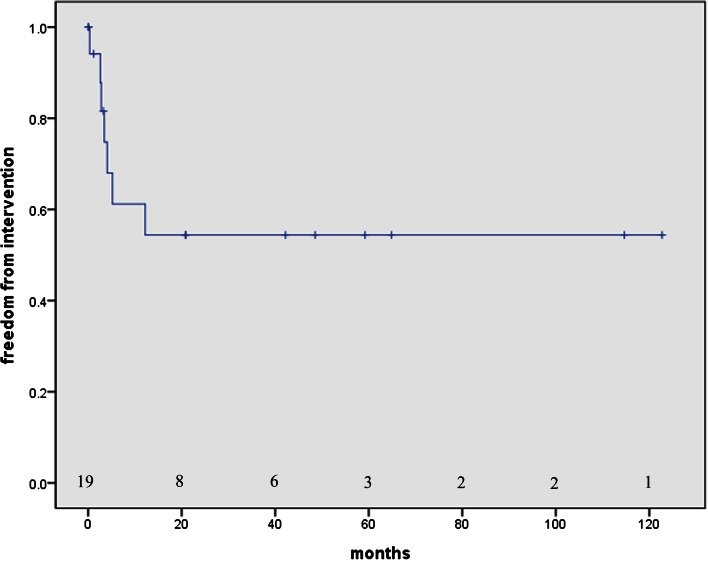
Freedom from intervention

Seven patients required postoperative percutaneous interventions; 1 required aortic recoarctation, 4 required interventions for branch pulmonary artery stenosis (right 1, left 3), and 2 required interventions for pulmonary valvar stenosis. All but one patient who underwent balloon valvuloplasty exhibited significant improvements following the percutaneous interventions. The patient who did not improve following the percutaneous intervention underwent re-operation. The freedoms from intervention were 81.6, 61.2, and 54.4 % at 3 months, 1, and 5 years, respectively (Fig. 2).

One patient underwent a re-operation. This patient was 5 days old, weighed 3.67 kg, and was TBA, type B IAA, and with an aberrant right subclavian artery and a very small aortic annulus. He had a side-by-side relationship of the great arteries and a 2LCx; 2R coronary anatomy. The left coronary artery had a retropulmonary pathway. We initially attempted balloon valvuloplasty to relieve his pulmonary valvar stenosis at the age of 89 days but failed.

The patient underwent transannular patch enlargement of the pulmonary valve and left pulmonary artery patch angioplasty at the age of 4.1 months. His most recent RVOT pressure gradient was approximately 25 mmHg with mild to moderate regurgitation.

There were no patients who exhibited a moderate or more severe degree of neoaortic valve regurgitation. There were 3 patients who had RVOT pressure gradients over 30 mmHg (3/16). All patients exhibited good clinical statuses (NYHA class I) without evidence of significant aortic restenosis.

## Discussion

AAO is present in only 5–9 % of TGA patients, but it is more frequent in TBA patients and occurs in more than 50 % of these patients [1, 10, 17]. The treatment of the combined transposition complexes with AAO is somewhat complicated. A two-staged repair has been adopted for these patients in the past. However, this approach had several disadvantages. Complete relief of arch obstruction through the thoracic approach is usually not possible due to diffuse arch hypoplasia, and pulmonary artery banding can produce relatively high incidences of postoperative neoaortic regurgitation and systemic ventricular dysfunction [8].

Some studies have shown the superiority of the one-stage repair over the two-stage repair. In 1993, Planche et al. [11] reported better results with a single-stage repair in a comparison of their experiences with both repair strategies. Since then, there has been a growing trend to adopt the single repair strategy as the treatment of choice for patients with TGA or TBA with AAO [3, 18]. The advantages of single-stage repairs over multiple-staged repairs are well known and include the early re-establishment of the normal physiologic circulation and the avoidance of the adverse effects of pulmonary artery banding. Diffuse arch hypoplasia or interruption is efficiently dealt with via median sternotomy at the time of the ASO. It is generally possible to perform a direct anastomosis for the interruption or coarctation with mild tension on the anastomosis [4]. However, some centers have continued to use multiple-staged repairs for these anomalies even in the recent era due to concerns about postoperative RVOT obstruction and the long complex procedure of the one-stage repair.

We believe that the most important advantage of one-stage repair over multiple-staged repair is relatively low postoperative mortality, although there is some risk of right ventricular outflow obstruction with the one-stage operation.

Planche et al. [11] showed that a two-staged repair group of 26 patients exhibited an actuarial survival rate at 5 years of 57.5 %. In contrast, a single-staged repair group of 14 patients exhibited an actuarial survival at 3 years of 78.5 %. Thus, these authors concluded that single-staged repairs of TGAs, VSDs, and AAOs provide better results than two-staged repairs. The overall mortality of the single-stage repair has been reported to be between 19 and 22 % [5, 10, 11, 14]. The mortality in our study was not significantly different from those reported in other studies, and there have been no early or late mortalities since 2006 among the 16 patients.

To perform the single-stage repair successfully, accurate coronary artery transfer is required. In many series, this procedure remained a major challenge in cases with unusual coronary artery anatomies. Pocar et al. [12] reported that a complex coronary anatomy remained an important risk factor for a difficult group of patients. However, in our study, we observed no mortality or morbidity related to coronary artery problems. We transferred the coronary arteries after the neoaorta reconstructions. Our coronary re-implantation technique ensured the very accurate selection of the coronary transfer site. Moreover, we believe that this approach is more effective in situations with significant size mismatches of the great arteries or unusual coronary artery patterns [2].

Huber et al. [4] used the pulmonary homograft patch enlargement of the aortic arch. These authors reported that 9 of 22 patients required the re-interventions for recurrent aortic coarctation. Eight patients underwent percutaneous intervention therapy, and 3 of these patients required surgical repair. One of our patients exhibited re-coarctation that was effectively managed by balloon aortoplasty. There were no re-operations for re-coarctation. Many patients with the combination of transposition complexes and AAO have small aortic annuli and narrow right ventricular outflow tracts. This situation can produce postoperative neopulmonary valvar stenosis and right ventricular outflow obstruction [6, 12]. Moreover, the anatomic factors that have previously been reported to be related to the occurrence of subneopulmonary stenosis are associated with AAO, coronary anomalies, side-by-side great artery relationships and the preoperative presence of organic subaortic stenosis [15]. However, Soszyn et al. [16] reported that there were no significant risk factors in their study. In our cases, 2 patients underwent RVOT patch enlargement for the relief RVOTO. Two of our patients exhibited RVOTO postoperatively, and this incidence was similar to that of Soszyn’s study because our cases consisted primarily of Taussig–bing anomalies with AAO. One patient was managed with balloon valvuloplasty, but another one patient with a type B interruption of the aortic arch and an aberrant right subclavian artery underwent re-operation with transannular patch enlargement of the RVOT. It is well known that an aberrant right subclavian artery is one of the risk factors for right ventricular outflow obstruction [7].

The postoperative intervention rate for this group of our patients was relatively high. The freedom from intervention was 54.4 % at 5 years. The main cause of intervention in our series was related to the peripheral pulmonary arteries. Four patients with branch pulmonary artery stenosis were treated with balloon angioplasty, and all of these patients improved. We believe that this result was basically related to technical problems during the arterial switch operations. Furthermore, we believe that the relatively high intervention rate was related to the low threshold for intervention in our hospital.

Mohammadi et al. [8] reported a relatively high incidence of re-operations for aortic valve regurgitation. These authors revealed that transpulmonary VSD closure is a significant risk factor for aortic valve regurgitation in all periods. Moreover, the discrepancy in the sizes of the arteries size was found to be highly associated with aortic valve regurgitation. Significant aortic valve regurgitation was significantly less frequent among the patients who were managed with homograft patches or the V-shaped resection technique. In our series, we closed the VSD through the tricuspid valve in 17 patients and through the tricuspid valve with an RV incision in 2 patients. We believe that it is possible to close VSDs through the tricuspid valve in nearly all cases. We make small incisions at the RVOT in cases of right ventricular outflow obstructions and occasionally excise or shave hypertrophic conal septum.

When we performed the neoaortic arch reconstructions, we used glutaraldehyde-treated autologous pericardial patches that were adjusted for the size of the large proximal main pulmonary artery. In our series, there were no patients who exhibited more than a mild degree of aortic valve regurgitation, and no patients have required re-operation for neoaortic valve regurgitation thus far. We believe that the low incidence of significant postoperative aortic valve regurgitation in our series might be related to our coronary transfer technique in which the coronary buttons are re-implanted in the reconstructed neoaorta as high as possible to prevent interruption of sinotubular junction geometry.

## Conclusions

In conclusion, one-stage repairs can be performed for patients with TGA or TBA and AAO with acceptable survival rates, and the midterm results are excellent, although postoperative catheter interventions are frequently required.

## References

[1] Aoki M, Forbes SJM, Jonas RA, Mayer JE, Castaneda AR (1994). Result of biventricular repair for double outlet right ventricle. J Thorac Cardiovasc Surg.

[2] Chang YH, Sung SC, Lee HD, Kim S, Lee YS (2005). Coronary reimplantation after neoaortic reconstruction can yield better result in arterial switch operation: comparison with open trap door technique. Ann Thorac Surg.

[3] Comas JV, Mignosa C, Cochrane AD, Wilkinson JL, Karl TR (1996). Taussig-bing anomaly and arterial switch: aortic arch obstruction does not influence outcome. Eur J Cardiothorac Surg.

[4] Huber C, Mimic B, Oswal N, Sullivan I, Kostolny M, Elliott M (2011). Outcomes and re-interventions after one-stage repair of transposition of great arteries and aortic arch obstruction. Eur J Cardiothorac Surg.

[5] Karl TR, Sano S, Brawn W, Mee RB (1992). Repair of hypoplastic or interrupted aortic arch via sternotomy. J Thorac Cardiovasc Surg.

[6] Lacour-Gayer F, Serraf A, Galletti L, Bruniaux J, Belli E, Piot D, Touchot A (1997). Biventricular repair of conotruncal anomalies associated with aortic arch obstruction: 103 patients. Circulation.

[7] McCrindle BW, Tchervenkov CI, Konstantinov IE, Williams WG, Neirotti RA, Jacobs ML (2005). Risk factors associated with mortality and interventions in 472 neonates with interrupted aortic arch: a Congenital Heart Surgeons Society Study. J Thorac Cardiovasc Surg.

[8] Mohammadi S, Serraf A, Belli E, Aupecle B, Capderou A, Lacour-Gayet F, Martinovic I (2004). Left-sided lesions after anatomic repair of transposition of the great arteries, ventricular septal defect, and coarctation: surgical factors. J Thorac Cardiovasc Surg.

[9] Parr GV, Waldhausen JA, Bharati S, Lev M, Whitman V (1983). Coarctation in Taussig-Bing malformation of the heart. Surgical significance. J Thorac Cardiovasc Surg.

[10] Pigott JD, Chin AJ, Weinberg PM, Wagner HR, Norwood WI (1987). Transposition of the great arteries with aortic arch obstruction. Anatomical review and report of surgical management. J Thorac Cardiovasc Surg.

[11] Planche C, Serraf A, Comas JV, Lacour-Gayet F, Bruniaux J, Touchot A (1993). Anatomic repair of transposition of the great arteries with ventricular septal defect and aortic arch obstruction. One stage versus two stage procedure. J Thorac Cardiovasc Surg.

[12] Pocaar M, Villa E, Degandt A, Mauriat P, Pouard P, Vouhe PR (2005). Long-term results after primary one-stage repair of transposition of the great arteries and aortic arch obstruction. J Am Coll Cardiol.

[13] Quaegebeur JM, Jonas RA, Weinberg AD, Blackstone EH, Kirklin JW, the Congenital heart surgeons society (1994). Outcomes in seriously ill neonates with coarctation of the aorta. J Thorac Cardiovasc Surg.

[14] Sandhu SK, Beekman RH, Mosca RS, Bove EL (1995). Single stage repair of aortic arch obstruction and associated intracardiac defects in the neonate. Am J Cardiol.

[15] Sinzobahamvya N, Blaschczok HC, Asfour B, Arenz C, Jussli MJ, Schindler E (2007). Right ventricular outflow tract obstruction after arterial switch operation for the Taussig-Bing heart. Eur J Cardiothoac Surg.

[16] Soszyn N, Fricke TA, Wheaton GR, Ramsay JM, d’Udekem Y, Brizard CP (2011). Outcomes of the arterial switch operation in patients with Taussig-bing anomaly. Ann Thorac Surg.

[17] Tchervenkov CI, Marelli D, Beland MJ, Gibbons JE, Paquet M, DObell ARC (1995). Institutional experience with a protocol of early primary repair of double outlet right ventricle. Ann Thorac Surg.

[18] Tchervenkov CI, Tahta SA, Cecere R, Beland MJ (1997). Single-stage arterial switch with aortic arch enlargement for transposition complexes with aortic arch obstruction. Ann Thorac Surg.

[19] Vouhe PR, Triquet F, Lecompte Y, Vernant F, Roux PM, Touati G (1988). Aortic coarctation with hypoplastic aortic arch. J Thorac Cardiovasc Surg.

